# CRISPR/Cas9 System as an Agent for Eliminating Polyomavirus JC Infection

**DOI:** 10.1371/journal.pone.0136046

**Published:** 2015-09-11

**Authors:** Hassen S. Wollebo, Anna Bellizzi, Rafal Kaminski, Wenhui Hu, Martyn K. White, Kamel Khalili

**Affiliations:** Department of Neuroscience, Temple University School of Medicine, Philadelphia, PA, 19122, United States of America; University of Nebraska—Lincoln, UNITED STATES

## Abstract

Progressive multifocal leukoencephalopathy (PML) is a fatal demyelinating disease of the central nervous system (CNS) caused by reactivation of the human polyomavirus JCV gene expression and its replication in oligodendrocytes, the myelin producing cells in the brain. Once a rare disease seen in patients with lymphotproliferative and myeloproliferative disorders, PML has been seen more frequently in HIV-1 positive/AIDS patients as well as patients undergoing immunomodulatory therapy due for autoimmune disorders including multiple sclerosis, rheumatoid arthritis, and others. As of now there is no cure for PML and in most cases disease progression leads to death within two years. Similar to other polyomaviruses, the JCV genome is small circular double stranded DNA that includes coding sequences for the viral early protein, T-antigen, which is critical for directing viral reactivation and lytic infection. Here, we employ a newly developed gene editing strategy, CRISPR/Cas9, to introduce mutations in the viral genome and, by inactivating the gene encoding T-antigen, inhibit viral replication. We first used bioinformatics screening and identified several potential targets within the JCV T-antigen gene that can serve as sites for the creation of guide RNAs (gRNAs) for positioning the Cas9 nuclease on the designated area of the viral genome for editing. Results from a series of integrated genetic and functional studies showed that transient or conditional expression of Cas9 and gRNAs specifically targets the DNA sequences corresponding to the N-terminal region of T-antigen, and by introducing mutation, interferes with expression and function of of the viral protein, hence suppressing viral replication in permissive cells. Results from SURVEYOR assay revealed no off-target effects of the JCV-specific CRISPR/Cas9 editing apparatus. These observations provide the first evidence for the employment of a gene editing strategy as a promising tool for the elimination of the JCV genome and a potential cure for PML.

## Introduction

The human neurotropic polyomavirus, JC (JCV) is the etiological agent of the fatal demyelinating disease progressive multifocal leukoencephalopathy (PML). Lytic infection of JCV in glial cells of the central nervous system (CNS) results in the death of oligodendrocytes, the cells that are responsible for the production of myelin sheaths in the brain, leading to a broad range of mild to severe neurological disturbances and eventually death [1]. There are a number of predisposing factors to PML but all involve some level of impairment of the immune system. While the disease was first recognized as a rare disorder predominantly seen in patients with lymphoproliferative and myeloproliferative disorders [2], the onset of the AIDS pandemic in the 1980s greatly increased the incidence of PML. It is now well-established that HIV-1 infection and AIDS remain the most frequent immunodeficiency setting for the reactivation of JCV, accounting for approximately 80% of PML cases [3]. In the 2000s, patients undergoing treatment for autoimmune disorders such as multiple sclerosis and rheumatoid arthritis with new therapeutic immunomodulatory monoclonal antibodies, including natalizumab [4], efalizumab [5] and rituximab [6], led to this class of drugs being recognized as a predisposing factor for PML. Immunosuppressive therapy given to transplant recipients is also a predisposing factor for PML [7].

JCV is a member of the polyomavirus family of viruses whose genome is comprised of double-stranded circular DNA of 5.1 kb in size, which produce two classes of proteins at the early phase of viral infection, i.e. before DNA replication, and the late phase of the infection cycle [8]. A bi-directional coding sequence positioned between the early and late genes is responsible for viral gene expression and contains the origin of viral DNA replication. The viral early protein, large T-antigen (T-Ag) and a family of smaller sized T-Ag proteins are produced by alternative splicing and have a regulatory role in orchestrating the virus during its replication cycle. The large T-antigen, in particular, is responsible for initiation of viral DNA replication and stimulation of viral late gene transcription, and thus is critical for all aspects of the viral life cycle. In addition, JCV T-Ag exhbits transforming ability in cell culture and its expression in animal models, in the absence of the other viral proteins, induces tumors of neural origin (for review see [25]). The late proteins are the viral capsid proteins VP1, VP2, and VP3 and a small regulatory protein known as agnoprotein [9]. Seroepidemiological studies have shown that JCV infection is very common in populations throughout the world and initial infection usually occurs during childhood [10]. The high seroprevalence of JCV infection and the rarity of PML suggest that the immune system is able to maintain the virus in a persistent asymptomatic state, since altered immune function appears to underlie all conditions that predispose to PML.

A number of treatment options have been applied to PML, largely without success [3]. Different approaches have targeted various points in the viral life cycle such as entry and replication. Since interaction between JCV and the serotonin 2A receptor (5-HT2AR) has been reported to be required for viral entry [11], risperidone, which binds 5HT2AR, has been studied but found to have no effect [12]. Small molecule inhibitors of viral replication such as cidofovir have been tested in vitro and in vivo but have yielded conflicting results [13, 14]. More recent studies with large cohorts of PML patients showed no impact of cidofovir on survival and an increased risk of neurological disability [3].

Clearly alternative strategy options are urgently required for treatment of this fatal demyelinating disease. New strategies that target the JCV viral genome for eradication are particularly attractive as this strategy can effectively target both actively replicating virus as well as persistent virus where the virus remains in a dormant state or where viral proteins are either not expressed or are produced at very low levels. Recent advances in engineered nuclease technology have raised the prospect that this therapeutic strategy may soon be possible in the clinic. Examples are zinc-finger nucleases (ZFN), transcription activator-like effector nucleases (TALEN) and more recently clustered regulatory interspaced short palindromic repeat (CRISPR)-associated 9 (Cas9) [15]. In particular, tools and techniques based on CRISPR/Cas9 offer unprecedented control over genome editing [16–19]. The CRISPR/Cas9 system was developed from the adaptive immune system of bacteria and archaea, and uses a short guide RNA (gRNA) to direct degradation of specific nucleic acids [20]. In principle, eradication of viruses from cells by CRISPR/Cas9 should be applicable to any DNA or RNA viruses with a DNA intermediate in its life cycle. For example, CRISPR/Cas9 has been used to inactivate the oncogenic human papilloma genes E6 and E7 in cervical carcinoma cells [21] and to facilitate clearance of intrahepatic hepatitis B genome templates *in vivo* [22]. More recently, we reported the use of CRISPR/Cas9 to eliminate HIV-1 provirus from latently infected cells and prevent new HIV-1 infection [23]. We now report the employment of CRISPR/Cas9 to to suppress JCV replication by deleting segments of the viral genome corresponding to coding regions of the T-Ag. As noted above, T-Ag, in addition to its critical role in JCV replication, binds to several cellular proteins such as p53 and pRb, and dysregulates proliferation of cells, thus potentially leading to transformation of cells and formation of tumors in several animal models [24, 25]. These properties provide an excellent opportunity to examine ablation of the JCV early genome by CRISPR/Cas9 in in vitro and in vivo models. Our results demonstrate that inactivation of T-Ag expression by gene editing can severely affect viral replication and possible its oncogenic activty in laboratory cells.

## Materials and Methods

### Cell culture and plasmids

The human oligodendroglioma cell line TC620 [26] and SVG-A, a cell line derived from primary human fetal glial cells transformed by origin-defective SV40 that expresses SV40 T-Ag [27], were maintained in Dulbecco’s Modified Eagle’s Medium (DMEM) supplemented with 10% fetal bovine serum (FBS) as we have previously described [26]. HJC-2 is a JCV-induced hamster glioblastoma cell line that expresses JCV T-Ag [28] and BsB8 is a mouse cell line derived from a tumor of cerebellar neuroectodermal origin arising in transgenic mice expressing the JCV early protein T-Ag [29]. Derivatives of SVG-A cells expressing Cas9 and JCV T-antigen gRNAs were developed by transfecting SVG-A cells with the pX260-derived plasmids (described below), selection in puromycin containing media, and isolation of single clones by dilution cloning.

### Plasmid Preparation

Vectors containing the human Cas9 and gRNA expression cassette, pX260, and pX330 (Addgene) were used to create various constructs. Both vectors contain a humanized Cas9 coding sequence driven by a CAG promoter and a gRNA expression cassette driven by a human U6 promoter [30]. The vectors were digested with BbsI and treated with phosphatase. A pair of oligonucleotides for each targeting site was annealed, phosphorylated and ligated to the linearized vector.

For TM1, these were:

TM1F: 5’–aacaaatgcaaagaactccaccctgatgaaggtggt– 3’

TM1R: 5’–taaaaccacctttatcagggtggagttctttgcattt– 3’,

For TM2, these were:

TM2F: 5’–aacgatgaatgggaatcctggtggaatacatttaatgagaagtgt– 3’

TM2R: 5’–taaaacacttctcattaaatgtattccaccaggattcccattcatc– 3’,

For TM3, these were:

TM3F: 5’–aaacaaggtactggctattcaaggggccaatagacaggt– 3’

TM3R: 5’–taaaacctgtctattggccccttgaatagccagtacctt– 3’.

The reporter construct, JCV_L_–LUC [26] and the pcDNA 3.1-large T-Ag expression plasmid have been previously described [31]. For lentivirus production, the following plasmids were obtained from Addgene: pCW-Cas9 (#50661), psPAX2 (#12260), pCMV-VSV-G (#8454), pKLV-U6gRNA(BbsI)-PGKpuro2ABFP (#50946), pMDLg/pRRE (#12251), pRSV-Rev (#12253). To construct the gRNA lentiviral expression plasmids for each of the three targets, the U6 expression cassette from each of the three pX330 gRNA plasmids described above was amplified by PCR with flanking primers (5’-tatgggcccacgcgtgagggcctatttcccatgattcc-3’ and 5’-tgtggatcctcgaggcgggccatttaccgtaagttatg-3’) and the PCR products treated with MluI and BamHI and cloned into pKLV-U6gRNA(BbsI)-PGKpuro2ABFP that had been cut with MluI and BamHI. The pCR 4-TOPO TA vector was from Life Technologies, Inc., Carlsbad, CA.

### Transient transfection and reporter assays

Co-transfection of JCV_L_-LUC reporter plasmid and T-Ag expression plasmid was performed as previously described [26, 32]. Briefly, TC620 cells were transfected with either reporter constructs alone (200 ng) or in combination with the various expression plasmids for 48h prior to harvesting. The total amount of transfected DNA was normalized with empty vector DNA. Luciferase assay was performed as previously described [26, 32].

### Production of clonal derivatives of SVG-A expressing Cas9 and gRNAs

SVG-A cells were transfected with pX260 or pX260-derived plasmids expressing each of the three gRNAs that are described above. Selection was done with 3 μg/ml puromycin and clones isolated by dilution cloning.

### Assay of JCV infection

Infection experiments were performed with SVG-A cells or the SVG-A clonal derivatives of SVG-A expressing Cas9 and gRNAs described above. Cells were infected with Mad-1 JCV at an MOI of 1 as we have previously described [33, 34], harvested and analyzed after 7 days together with uninfected control cultures. Expression of the viral proteins VP1 and agnoprotein was measured in whole cell protein extracts by Western blot. In parallel, the growth media of the cells was also collected to measure viral load by Q-PCR.

### Immunocytochemistry

TC620 cells were transfected with an expression plasmid for FLAG-tagged Cas9 and immunocytochemistry was performed with mouse anti-Flag M2 primary antibody (1:500, Sigma) as previously described [23].

### Analysis of T-Ag gene cleavage by PCR

BsB8 or HJC-2 cells were transfected with plasmid pX260 or pX260-derived plasmids expressing the gRNAs described above and total genomic DNA was extracted after 48 hours. The T-Ag gene was amplified by PCR using primers that flank the JCV T-Ag coding region (5’-gcttatgccatgccctgaaggt-3’ and 5’-atggacaaagtgctgaataggga-3’) and PCR products were subjected to agarose gel electrophoresis.

### Production of lentiviral vectors for Cas9 and creation of HJC-2 cells expressing inducible Cas9

To produce a lentiviral vector for transduction of doxycycline-inducible Cas9 expression, 293T cells were transfected with plasmids pCW-Cas9, psPAX2 and pCMV-VSV-Gusing the calcium phosphate precipitation method [35]. Lentivirus was harvested from the supernatant after 48 h, cleared by centrifugation and passed through a 0.45 μm filter as previously described [36]. To obtain stably transduced HJC-2 clonal cell derivatives inducibly expressing Cas9, lentivirus was added to HJC-2 cells in the presence of 6 μg/ml polybrene followed by selection with 3 μg/ml puromycin and isolation of clones by dilution cloning. For induction of Cas9 expression in the resulting clones, 2 μg/ml doxycycline was added to the culture media.

### Production of lentiviral vectors for gRNAs and transduction of HJC-2 cells expressing inducible Cas9

To produce a lentiviral vector for transduction of the three gRNAs, each of the three gRNA lentiviral expression plasmid derivatives constructed as described above from pKLV-U6gRNA(BbsI)-PGKpuro2ABFP were transfected into 293T cells by calcium chloride precipitation together with packaging plasmids pCMV-VSV-G, pMDLg/pRRE and pRSV-Rev. Lentivirus was harvested from the supernatant after 48h, cleared by centrifugation, passed through a 0.45 μm filter, and added to HJC-2 cells in the presence of 6 μg/ml polybrene followed by selection. After 24 hours, the transduced cells were treated with 2 μg/ml doxycycline to induce Cas9 expression and after another 48 hours harvested and analyzed for T-Ag mutations by PCR/sequencing, T-Ag expression by Western blot, and clonogenicity by colony formation assay.

### Analysis of InDel mutations

Since the cleavage of DNA by Cas9 leaves behind characteristic short insertion/deletion (InDel) mutations, we analyzed the sequence of the T-Ag gene from the gRNA-transduced HJC-2 cells. Total genomic DNA was isolated from cells using a genomic DNA purification kit according to the manufacturer’s instructions (5prime Inc., Gaithersburg MD) and the regions of the T-Ag gene that had been targeted were amplified by PCR using flanking primers. For TM1 and TM2, the following primers are used 5’-ctctggtcatgtggatgctgt-3’ and 5’- atggacaaagtgctgaataggga-3’. Primers 5’-gcttatgccatgccctgaaggt-3’ and 5’-acagcatccacatgaccagag-3’ were used for TM3. The PCR products were cloned into the TA cloning vector pCR4-TOPO and colonies sequenced.

### SURVEYOR assay

The presence of mutations in PCR products derived from HJC-2 cells expressing Cas9 and transduced by lentiviral vectors for gRNAs was examined using the SURVEYOR Mutation Detection Kit (Transgenomic) according to the manufacturer’s protocolusing the same primers as used for the InDel mutation analysis. The heterogeneous PCR product was denatured for 10 min at 95°C and then hybridized by gradual cooling using a thermocycler. Three hundred nanograms of hybridized DNA (9 μl) was digested with 0.25 μl of SURVEYOR Nuclease, which is a mismatch-specific DNA endonuclease used to scan for mutations in heteroduplex DNA, plus 0.25 μl SURVEYOR Enhancer S and 15 mM MgCl_2_ for 4 h at 42°C. Stop Solution was added and samples were resolved on a 2% agarose gel together with equal amounts of control samples treated in parallel but derived from HJC-2 cells expressing Cas9 but not transduced by lentiviral vector for gRNA. The SURVEYRO assay was also used to detect the presence of off-target mutations on cellular genes using stable derivates of SVG-A cell lines expressing Cas9 and gRNAs.

### Colony formation assay

HJC-2 cells expressing inducible Cas9 were transduced with lentiviral expression vectors for gRNAs for each of the three targets either alone or in combination. Cells were plated for colony formation assays in the presence or absence of doxycycline. Cells were grown for 10–14 days, washed with PBS and fixed and stained with 40% methanol and 0.4% methylene blue. Colonies with more than 50 cells were counted.

## Results

**Fig 1:**
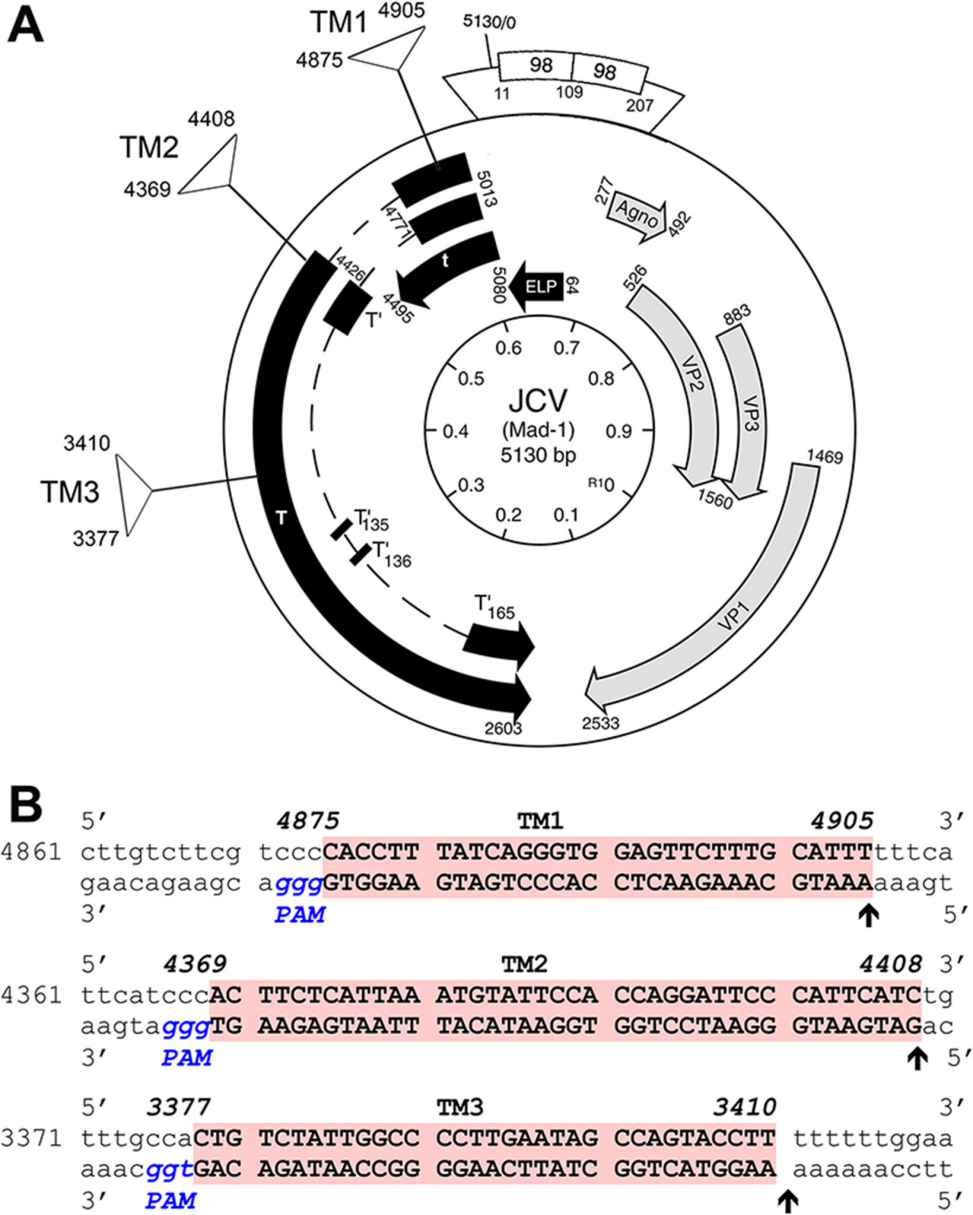
Design of guide RNAs for CRISPR/Cas9 targeting of JCV. **A.** Three gRNAs (TM1, TM2 and TM3) were designed at different positions within the coding region for JCV T-Ag (T) as shown. The T-Ag coding region begins at nucleotide (nt) 5013 of the 5130 nt circular Mad-1 JCV genome (NCBI Reference Sequence: NC_001699.1; Frisque et al, 1984) and proceeds counter clockwise to nt 2603. **B.** The sequence of the JCV genome at each of the three targeted sites (bold and red boxes) is given. Note that the sequence of the top strand is clockwise and antisense to the coding region of T-Ag since T-Ag is transcribed counter clockwise and shown on the bottom (sense strand). The position of the protospacer adjacent motif (PAM) sequences are shown in blue italics.

To target the JCV genome using the CRISPR/Cas9 system, we designed gRNAs for three different regions within the gene encoding T-Ag, named TM1, TM2 and TM3. The positions of these regions with respect to the JCV genome are shown in Fig 1A. In reference to the JCV Mad-1 strain, the DNA sequences corresponding to T-Ag coding region start at nucleotide 5013 (NCBI Reference Sequence: NC_001699.1 [37]) and proceed counter clockwise to nt 2603. The nucleotide composition of the JCV genome at each of the three target sites is shown in Fig 1B. Note that the sequence of the top strand is clockwise and complementary to the coding sequence of T-Ag, which is transcribed counter clockwise. The position of the protospacer adjacent motif (PAM) sequence is highlighted in blue. The sequence of the JCV genome at each of the three targeted sites are shown in bold and highlighted by red boxes.

**Fig 2:**
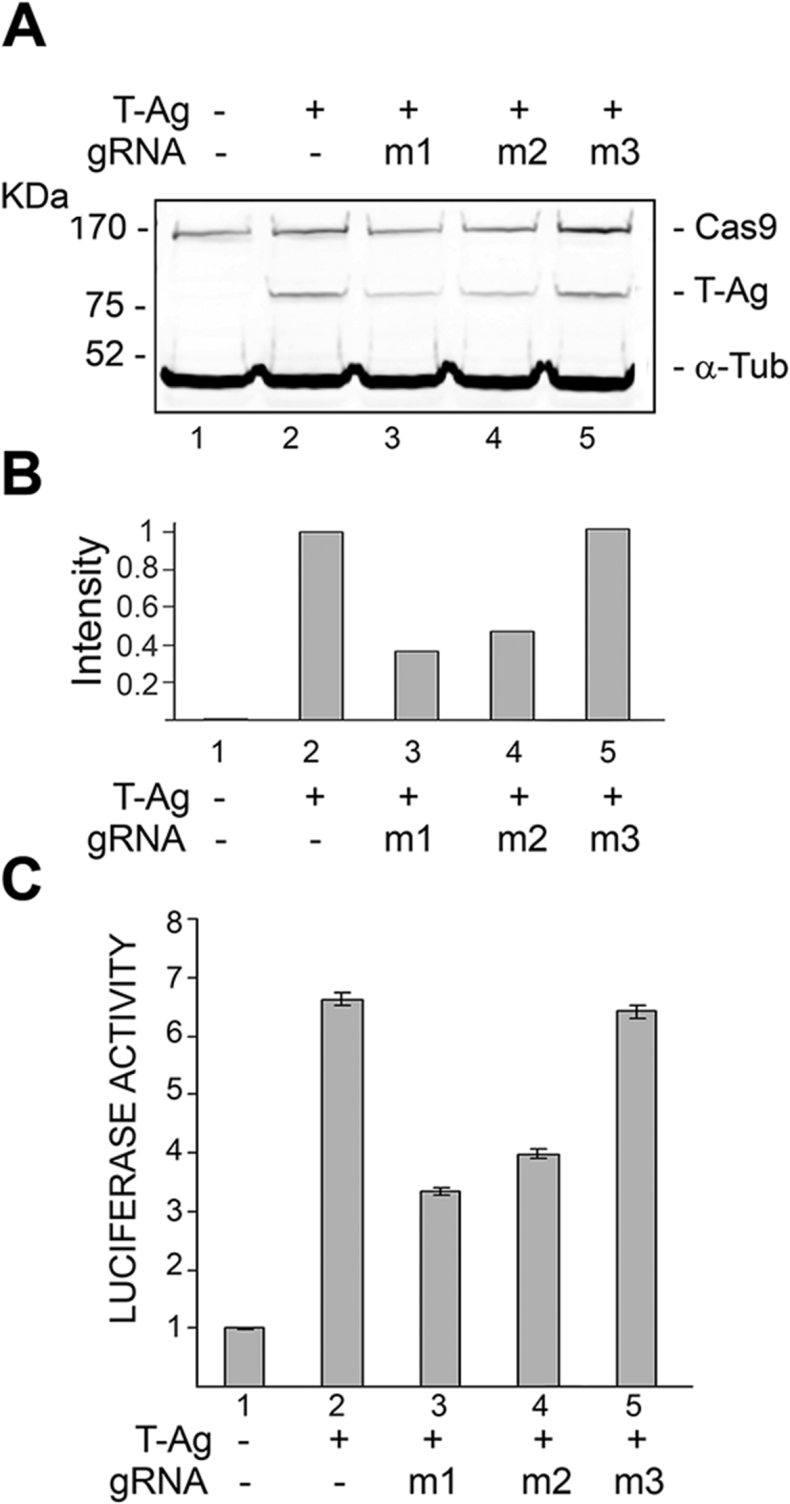
Expression of gRNAs m1 and m2 knocks down T-Ag expression and effect T-Ag-induced activation of the JCV late promoter. **A.** Cell extracts prepared in Fig 2A were analyzed by Western blot for expression of T-Ag, Cas9, and α-tubulin (loading control). **B.** The Western blots in Fig 2B were quantified using Bio-Rad Quantity One software and shown as a histogram normalized to T-Ag alone (lane 2). **C.** TC620 cells were transfected with JCV_L_-LUC reporter plasmid and expression plasmid for Cas9 with and without expression plasmids for T-Ag and each of the gRNAs shown in Fig 1 alone or in combination as indicated. Cells were harvested and luciferase activity was assayed as described in Materials and Methods. Activities were normalized to cells transfected with reporter plasmid alone (lane 1).

In the first set of experiments we investigated the ability of Cas9 along with the various gRNAs, i.e. TM1, TM2 and TM3, to suppress expression of T-antigen in the human oligodendrocytic cell line, TC620. Results from Western blot analysis of TC620 cells transfected with plasmid expressing T-antigen, either alone or in combination with Cas9 and/or in combination with T-Ag targeting gRNA expression plasmids are shown in Fig 2A. As seen in this figure, the presence of Cas9 together with either TM1 or TM2 gRNAs noticeably decreased the level of T-Ag production in the transfected cells. However, expression of gRNA TM3 showed no significant effect on the level of T-Ag expression. Fig 2B illustrates results from quantification of T-antigen production based on the intensity of the band corresponding to T-antigen, which was normalized to the housekeeping gene, β-tubulin. Next, we performed functional studies based on the ability of T-Ag to stimulate JCV late promoter activity [38]. TC620 cells are particularly appropriate in this study as their oligodendrocytic origin allows cell type-specific transcription of the JCV promoter at the basal level which can be induced upon T-Ag expression. Results from transcription studies using the JCV late promoter, JCV_L_, driving the reporter luciferase gene showed that the level of JCV_L_ promoter activation by T-Ag is significantly decreased in cells expressing Cas9/gRNA TM1 or Cas9/gRNA TM2, but not Cas9/gRNA TM3. These observations are in good agreement with the extent of the impact of TM1, TM2 and TM3 gRNAs on the level of expression of T-Ag thus indicating that targeting T-Ag as the gene editing strategy may have a functional consequence on subsequent events related to viral lytic infection.

**Fig 3:**
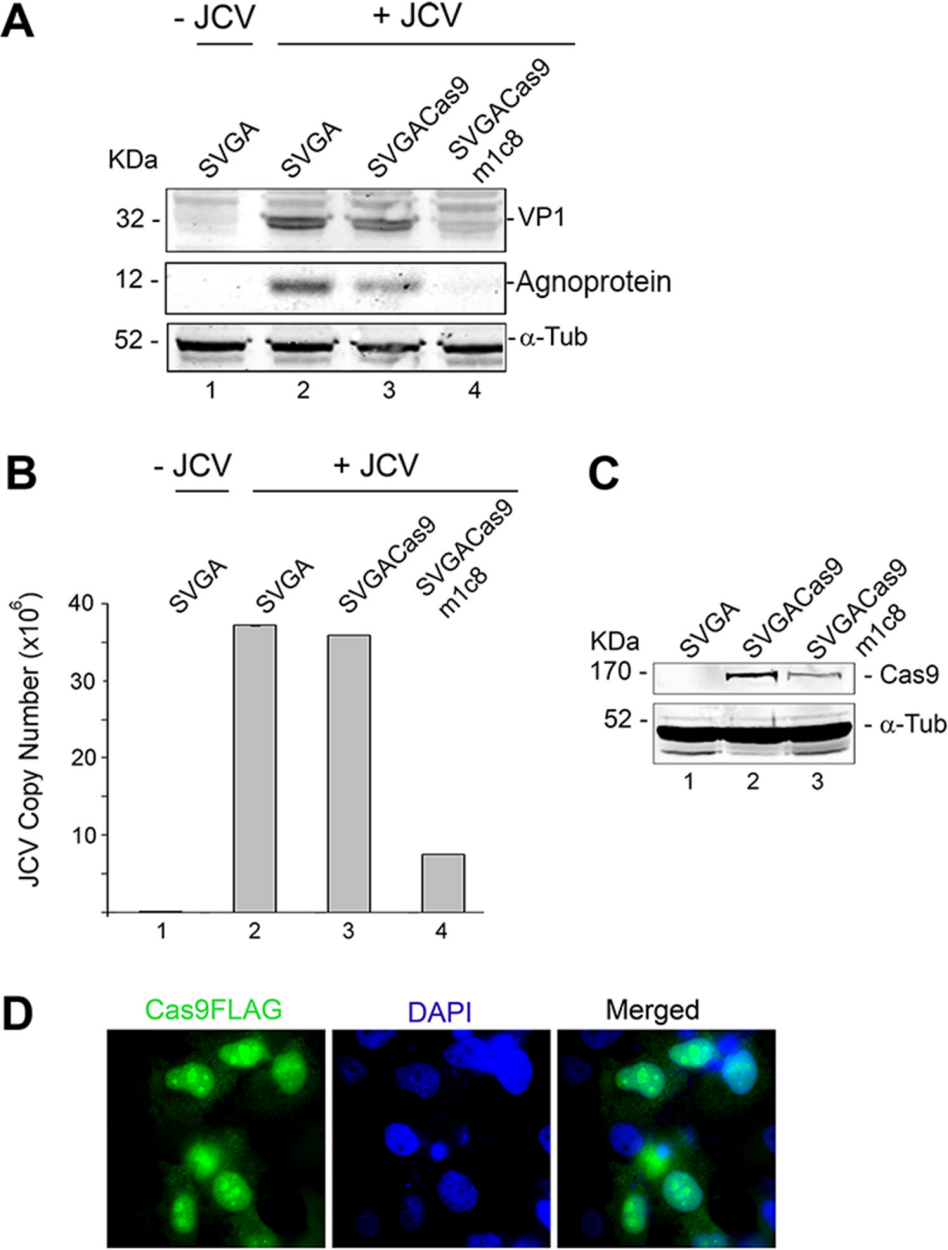
A clonal derivative of SVG-A expressing Cas9 and gRNA m1 has reduced capacity to support JCV infection. **A.** SVG-A cells were transfected with Cas9 or Cas9 plus gRNA m1 and stable clonal cell lines selected. Representative clones were selected and assayed for JCV infection (MOI = 1, 7 days post-infection): results from one clone with Cas9 alone and one clone with Cas9 plus gRNA m1 (c8) relative to parental SVG-A cells. Viral infection was assessed by Western blot for VP1 and agnoprotein with α-tubulin as a loading control. **B.** Viral loads in the culture supernatants from the experiment in Panel A were quantified using Q-PCR and are shown as copy number. **C.** SVG-ACas9 and SVG-ACas9m1c8 were assayed for Cas9 expression by Western blot with α-tubulin used as a loading control. **D.** TC620 cells were transfected with expression plasmid for FLAG-tagged Cas9 and immunocytochemistry performed with anti-FLAG antibody as described in Materials and Methods. Nuclei were labeled with 4',6-diamidino-2-phenylindole (DAPI).

To investigate the effects of CRISPR/Cas9 on JCV infection, we used the SVG-A cell line that supports both viral gene expression and allows for complete productive viral lytic infection. First, we developed stable clonal cells from SVG-A cells that express either Cas9 or Cas9 plus gRNA TM1. Three separate clones were selected and used for JCV infection at an MOI = 1.0 for seven days. Viral infection was assessed by Western blot for the presence of the viral capsid protein, VP1 and the auxiliary protein, agnoprotein with α-tubulin as a loading control. In parallel, we used quantitative PCR (Q-PCR) to determine the level of viral DNA in the culture media from the above-noted experiment as a marker of DNA replication and therefore virus production. As shown in Fig 3A, the clonal cell line expressing only Cas9 (lane 3) supported JCV infection as evidenced by the presence of the viral capsid protein, VP1 and the auxiliary agnoprotein in these cells (lane 2), whereas the SVG-A clone expressing both Cas9 plus gRNA TM1 failed to support viral replication. This finding was supported by Q-PCR experiments to measure virus in the culture supernatants (Fig 3B). It is, however, noteworthy to mention that some of the clonal cells, which were originally transfected with both Cas9 and gRNA TM1, were able to support JCV replication, suggesting that these cells either lost Cas9 expression and/or gRNA TM1 is no longer produced (data not shown). To show that JCV infection has no effect on Cas9 expression, Cas9 expression was confirmed by Western blot in SVG-A Cas9 and SVG-A Cas9m1c8 cells (Fig 3C) and FLAG-tagged Cas9 was shown by immunocytochemistry to localize to the nucleus (Fig 3D).

**Fig 4:**
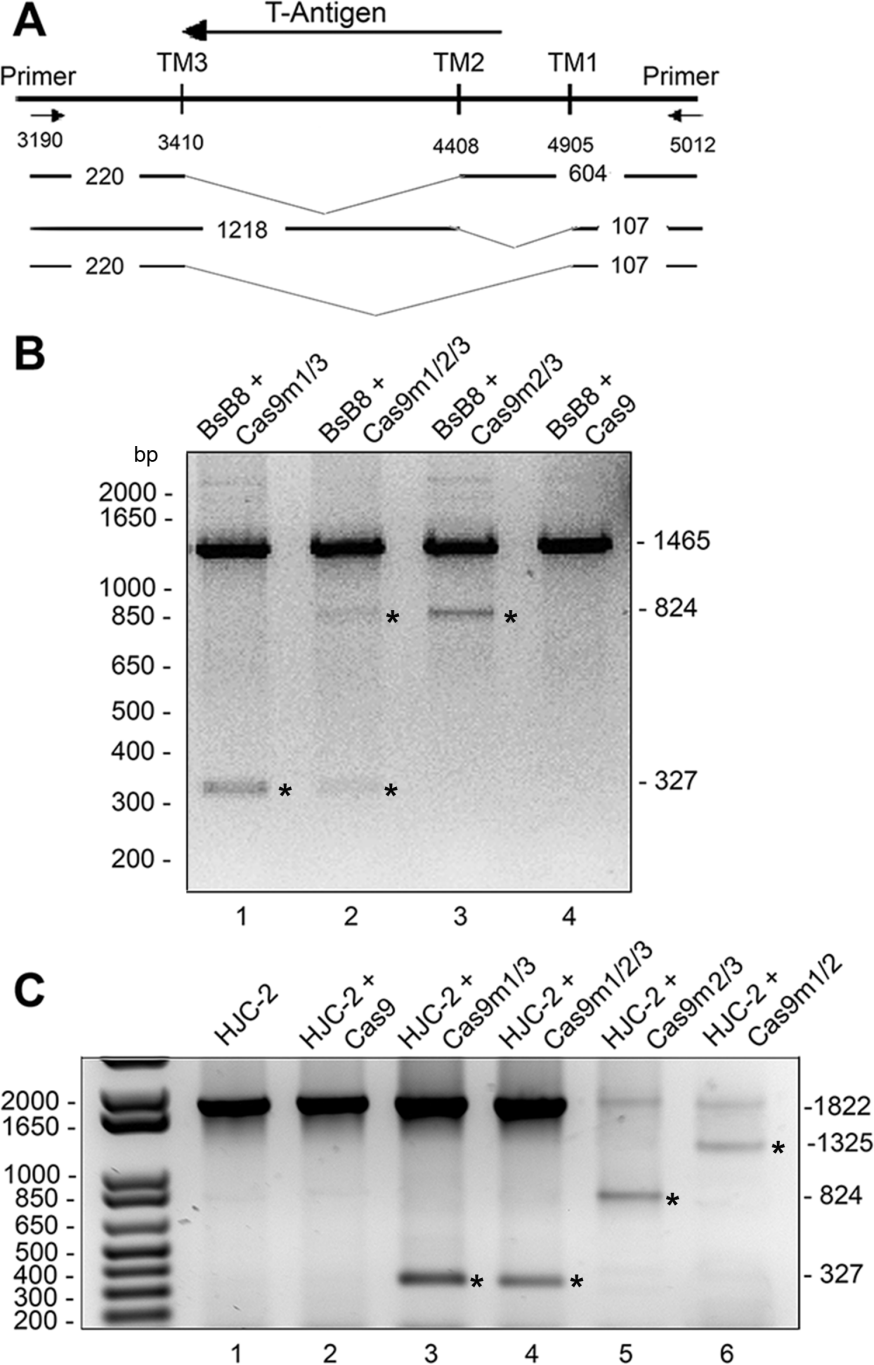
Direct demonstration of T-Ag gene cleavage after transient transfection of Cas9 and JCV-specific gRNA. Mouse BsB8 and hamster HJC-2 cells, which both contain integrated JCV T-Ag gene, were transfected with expression plasmids for Cas9 and the gRNAs in various combinations as indicated and genomic DNA amplified using JCV-specific primers. **A.** Diagram of the T-Ag gene indicating the positions of the PCR primers and the expected cleavage points and the expected lengths of resulting fragments of the T-Ag gene. **B.** The T-Ag gene from transfected BsB8 cells was amplified by PCR, electrophoresed on an agrose gel and labeled with ethidium bromide. **C.** The T-Ag gene from transfected HJC-2 cells was amplified by PCR, electrophoresed on an agrose gel and labeled with ethidium bromide. The images in panels B and C are inverted for clarity of presentation.

Next, we examined the ability of Cas9 and the gRNAs in editing the JCV DNA sequence corresponding to the T-Ag gene. For these experiments, we utilized BsB8 cells, a murine cell line that contains the JCV early region as an incorporated transgene and expresses T-Ag [29]. In addition, we used another cell line, HJC-2, a hamster cell line isolated by limiting dilution from a glioblastoma induced by intracerebral injection of JCV [28]. These cells also carry the JCV early genes integrated into the host genome and express T-Ag. These cell lines were chosen for the experiments because their integrated copies of the JCV genome allow a precise measurement of the editing capabilities of the Cas9/gRNAs targeting the JCV sequences. The cells were transfected with expression plasmids for Cas9 and the gRNAs in various combinations and genomic DNA was amplified using JCV-specific primers. Fig 4A illustrates the positions of the cleavage points and the expected lengths of the resulting DNA fragments corresponding to the T-Ag gene after editing. Results from DNA analysis by agarose gel electrophoresis are shown in Fig 4B and 4C, suggesting the appropriate cuts of the DNA by Cas9/gRNA based on the expected size of the DNA fragments after each transfection. For example, Cas9m1/m3 (Fig 4B, lane 1; Fig 4C, lane 3) has a band of 327 (220 + 107) consistent with a deletion in the T-Ag gene between m1 and m3. We also noticed a difference in the size of the integrated JCV DNA corresponding to the early region in the genomes of the BsB8 and HJC-2 cells, suggesting that truncated copies of JCV T-Ag DNA are incorporated in the BsB8 cells, which results in amplification of a smaller size of DNA. Nevertheless, this variation has no impact on the ability of our gene editing strategy to cleave the correct target sequences that are present in these cells.

**Fig 5:**
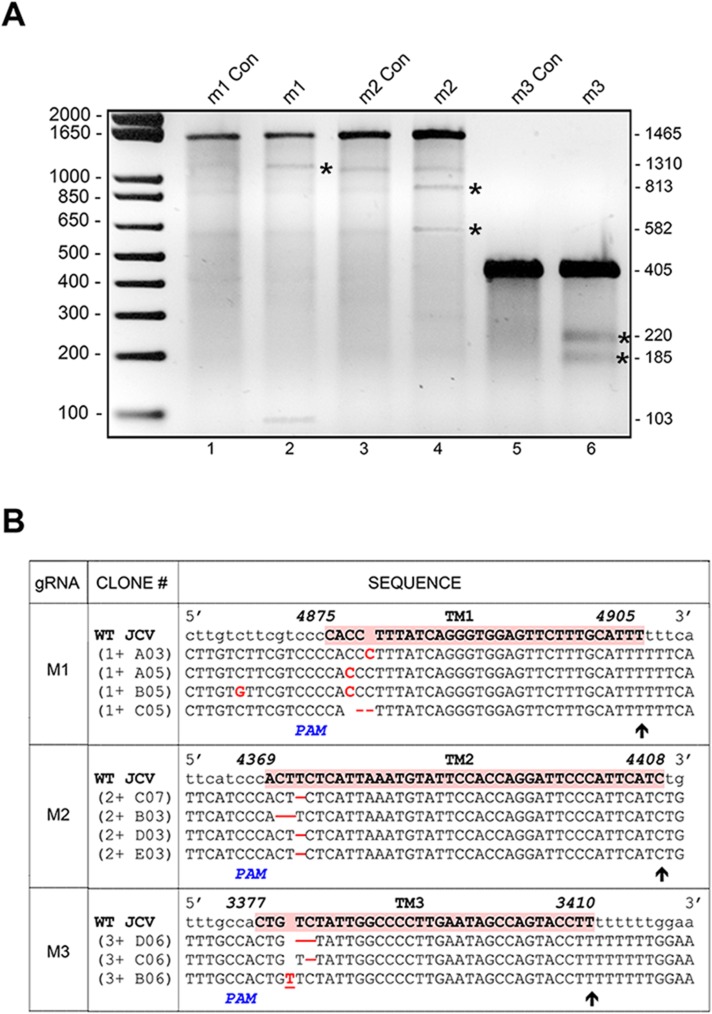
Stable derivatives of HJC-2 cells expressing doxycycline-inducible Cas9 show InDel mutations of the T-Ag gene upon transduction with lentiviruses expressing JCV-specific gRNAs and doxycycline induction. **A.** HJC-2 cells expressing doxycycline-inducible Cas9 were transduced with lentiviruses expressing JCV-specific gRNAs and treated with and without doxycycline as described in Materials and Methods. Total genomic DNA was extracted and regions of the T-Ag were amplified by PCR, cloned into pCR4-TOPOTA vector and sequenced. **B.** The Surveyor assay was used to detect the presence of mutations in PCR products derived from HJC-2 cells expressing Cas9 and transduced by lentiviral vectors for each of the gRNAs (m1, m2 and m3). PCR products were denatured and hybridized by gradual cooling as described in Materials and Methods. Hybridized DNA was digested with SURVEYOR nuclease to cut heteroduplex DNA and samples were resolved on a 2% agarose gel together with equal amounts of control samples treated in parallel but derived from HJC-2 cells expressing Cas9 but not transduced by lentiviral vectors endocing gRNAs (m1 Con, m2 Con, m3 Con).

**Fig 6:**
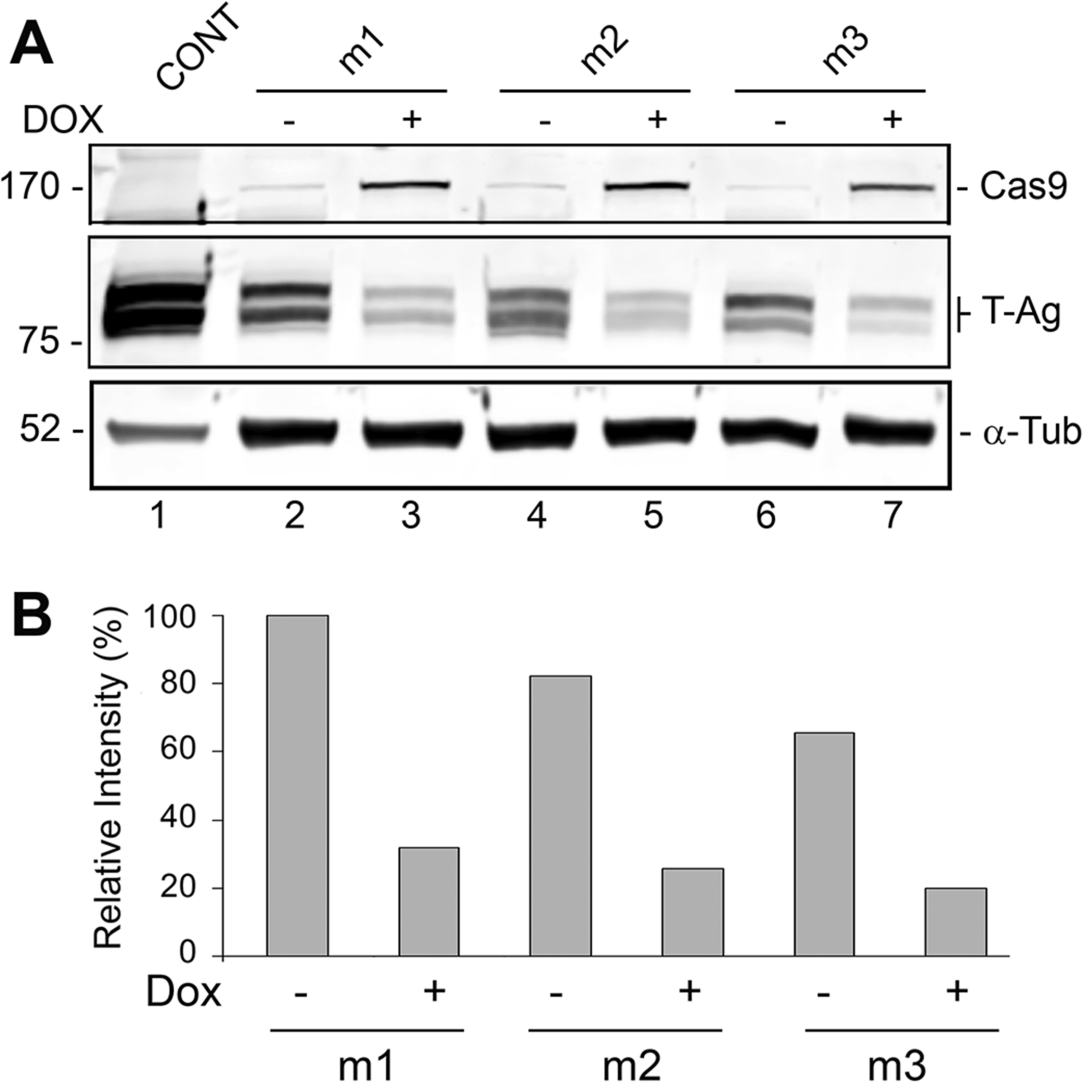
Stable derivatives of HJC-2 cells expressing doxycycline-inducible Cas9 show ablation of T-Ag expression upon transduction with lentiviruses expressing JCV-specific gRNAs. HJC-2 stable cell clones expressing doxycycline-inducible Cas9 were transduced with lentiviral vectors for each of the three gRNAs as described in Material and Methods. After 24 hours, the transduced cells were treated with and without 2 μg/ml doxycycline and after another 48 hours harvested and expression of T-Ag and Cas9 analyzed by Western blot with α-tubulin as a loading control.

**Fig 7:**
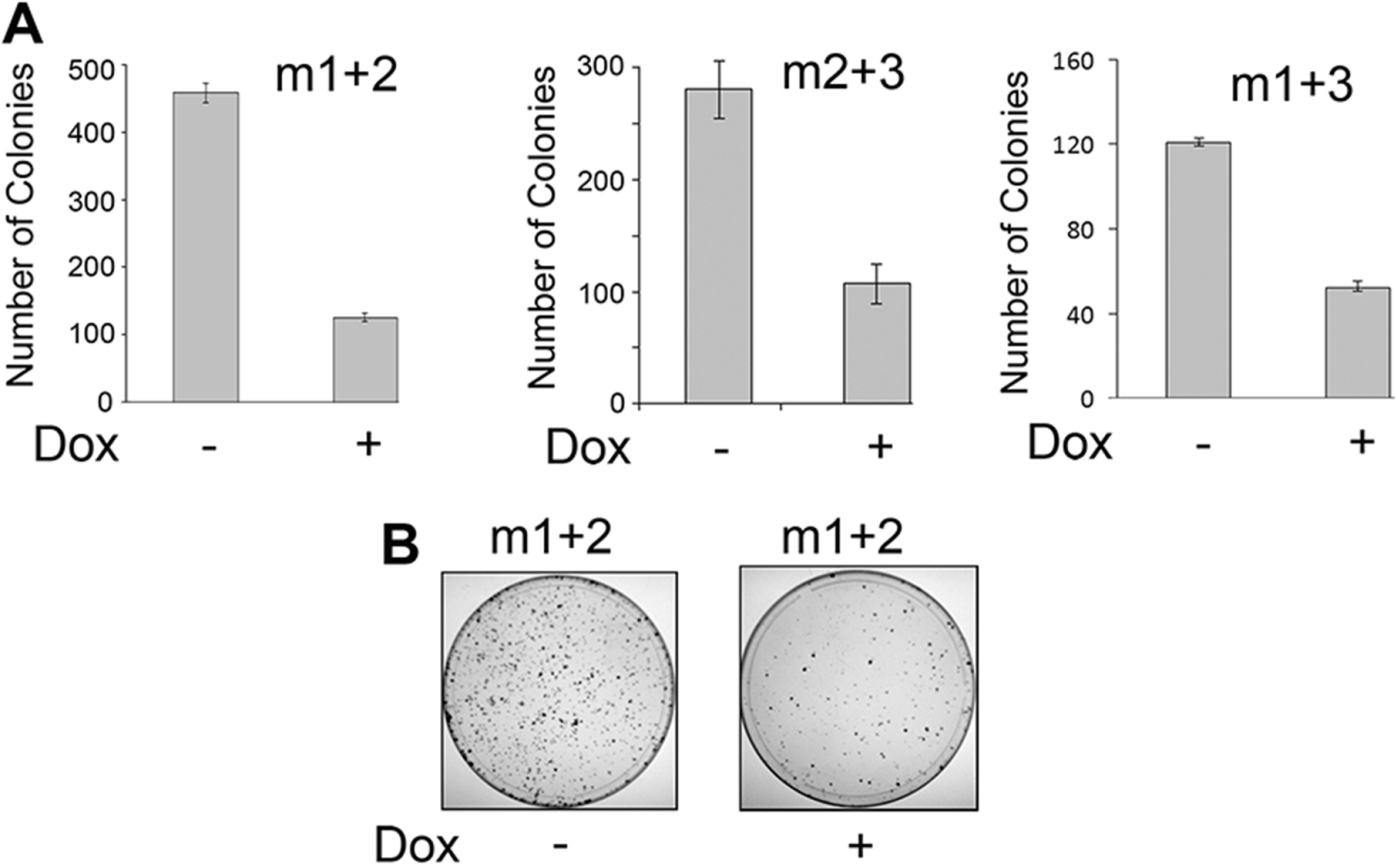
Stable derivatives of HJC-2 cells expressing doxycycline-inducible Cas9 show reduced colony formation upon transduction with lentiviruses expressing JCV-specific gRNAs. **A.** HJC-2 stable cell clones expressing doxycycline-inducible Cas9 were transduced with lentiviruses expressing m1, m2 and m3 gRNAs in various combinations as indicated, plated, treated with or without doxycycline, and assessed for colony formation as described in Materials and Methods. Results are shown as histograms of the total numbers of colonies obtained with the error bars representing one standard deviation calculated from replicate colony counts. **B.** Photograph of a representative experiment showing two dishes from panel A after methylene blue staining.

Next, to increase the delivery efficiency of Cas9 and gRNAs, we placed a doxycycline-inducible Cas9 gene in a lentiviral vector and used the construct to transduce HJC-2 cells for insertion/deletion (InDel) analysis within the JCV genome that could be caused by Cas9 and gRNAs m1, m2 and m3. InDel mutations are characteristic short mutations visually left behind by Cas9 cleavage of DNA. InDel mutations are detected in the corresponding region of the T-Ag gene of genomic DNA extracted from these cultures after 48h by sequencing of clones derived from PCR amplified products. Results from sequencing (Fig 5B) show that most of the InDels were close to the PAM regions and consisted of insertion or deletion of one or two nucleotides and thus would be predicted to cause frameshift mutations affecting T-Ag translation. As expected, when we examined T-Ag expression by Western blot, we found that T-Ag expression was ablated with each of the three gRNAs upon inducing Cas9 expression with doxycycline (Fig 6A). Fig 6B illustrates quantitative analysis of T-Ag expression upon induction of Cas9 by doxycycline and gRNA expression. We also investigated the effect of Cas9 expression together with gRNAS m1, m2 and m3 in various combinations on clonogenecity of HJC-2, a phenotype that relies on T-Ag. As seen in Fig 7, the induction of Cas9 expression by doxycycline alone with the gRNAs (as indicated) severely decreased the number of colonies that are formed by these cells (Panel A), indicative of T-Ag suppression by the Cas9/gRNAs in the cells. Fig 7B illustrates a typical colony formation assay.

**Fig 8:**
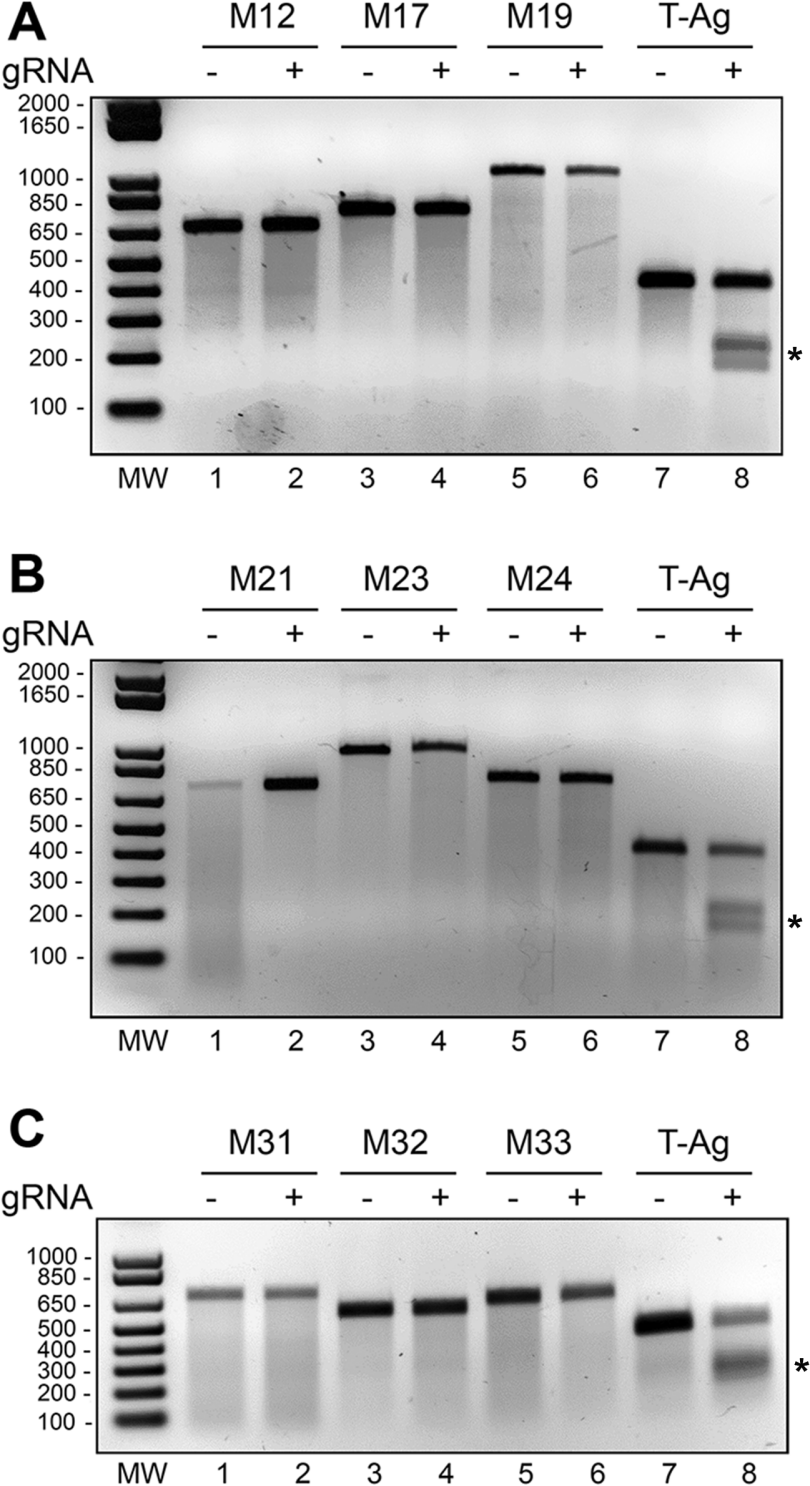
Stable derivatives of SVG-A cells expressing Cas9 and gRNAs show no InDel mutations in off-target genes. The SURVEYOR assay was used to detect the presence of mutations in PCR products derived from SVG-A cells expressing Cas9 and gRNAs. *m* 1 (A), m2 (B) and m3 (C). Human cellular genes with the highest degree of homology to each motif were identified by BLAST search at the NCBI website (http://www.ncbi.nlm.nih.gov/). For each motif, PCR product was amplified from the top three genes with the highest degree of homology and examined for InDel mutations using the SURVEYOR assay as described in Materials and Methods. Amplification of T-Ag was the positive control in each panel. **A.** For motif m1, we amplified M12 (NM_017821, human RHBDL2 rhomboid, veinlet-like 2 (Drosophila), Gene ID: 54933, NCBI Ref Seq: NC_000001.11, >gi|568815597:c38941830-38885806), M17 (NM_001243540, human KIAA1731NL, Gene ID: 100653515, NCBI Ref Seq: NC_000017.11, >gi|568815581:78887721–78903217) and M19 (NM_016252, human BIRC6 baculoviral IAP repeat containing 6, Gene ID: 57448, NCBI Ref Seq: NC_000002.12); **B.** For motif m2, we amplified M21 (NM_012090, human MACF1 microtubule-actin crosslinking factor 1, Gene ID: 23499, NCBI Ref Seq: NC_000001.11, >gi|568815597:39084167–39487138), M23 (NM_005898, human CAPRIN1 cell cycle associated protein 1, Gene ID: 4076, NCBI Ref Seq: NC_000011.10, >gi|568815587:34051683–34102610), M24 (NM_024562, human TANGO6 transport and Golgi organization 6 homolog (Drosophila), Gene ID: 79613, NCBI Ref Seq: NC_000016.10, >gi|568815582:68843553–69085482); **C.** For motif m3, we amplified M31 (NM_001048194, human RCC1 regulator of chromosome condensation 1, Gene ID: 1104, NCBI Ref Seq: NC_000001.11, >gi|568815597:28505943–28539196), M32 (NM_004673, human ANGPTL1 angiopoietin-like 1, Gene ID: 9068, NCBI Ref Seq: NC_000001.11, >gi|568815597:c178871353-178849535), M33 (NM_174944, human TSSK4 testis-specific serine kinase 4, Gene ID: 283629, NCBI Ref Seq: NC_000014.9, >gi|568815584:24205530–24208248).

Finally, to assess any possible off-target events occurring in the cellular genome, stable Cas9 and gRNA expressing SVG-A cells were analyzed by SURVEYOR assay for InDel mutations in off-target genes. Human cellular genes with the highest degree of homology to each of the three motifs were identified by BLAST search at the NCBI website (http://www.ncbi.nlm.nih.gov/). For each motif, PCR products were amplified from the top three genes with the highest degree of homology and examined for InDel mutations using the SURVEYOR assay as described in Materials and Methods (Fig 8). Motif 1 gRNA was analyzed in Panel A, motif 2 in Panel B, and motif 3 in Panel C. Amplification of T-Ag was used as a positive control in each panel and the presence of additional bands as a result of the cleavage by SURVEYOR nuclease are shown by asterisks. In all cases, no cleavage of the off-target genes was detected, indicating the specificity of the CRISPR/Cas9.

## Discussion

The public health significance of PML is high because individuals with many types of immune impairment are vulnerable and while the disease is rare, a broad range of people with impaired immune systems are at-risk including HIV-1/AIDS patients and patients receiving therapeutic immunomodulatory monoclonal antibodies for the treatment of autoimmune disorders such as multiple sclerosis and rheumatoid arthritis [3]. Since JCV is a DNA virus, targeted genome editing strategies may be considered as a potential tool to eliminate viral genomes. The CRISPR/Cas9 technology is versatile, straightforward, and rapid, and many clinical applications are anticipated especially in the fields of genetic diseases, cancer, and viral infections [16–18]. Here, we have investigated the potential of CRISPR/Cas9 for JCV elimination by targeting regions within the early gene encoding T-Ag, which is required for viral DNA replication and effecting changes in the intracellular environment necessary for the viral life cycle to proceed [24, 25]. The expression of Cas9 and JCV-specific gRNAs together in cells containing an integrated T-Ag gene showed that specific deletions could be generated. In transient transfection experiments, we found that expression plasmids for Cas9 together with JCV-specific gRNAs in single or in combination could knockdown T-Ag expression, as determined by Western blot, and T-Ag function, as measured by reduction of transactivation of JCV late promoter activity and its clonogenic activity in transformed cells. In the transformed human glial cell line SVG-A, which supports JCV replication, derivation of clonal cell lines that express Cas9 and JCV-specific gRNAs revealed clones that had become refractory to JCV infection. Note that these cells express SV40 T-Ag, which is not targeted by the JCV-specific gRNAs, indicating that JCV T-Ag is necessary for JCV infection even when SV40 T-Ag is supplied *in trans*.

In the transformed hamster cell line HJC-2, which contains at least one copy of JCV stably integrated into the genome and expresses JCV T-Ag, transduction with lentivirus for Cas9 and gRNA induced InDel mutations in the T-Ag coding sequence and thus ablated T-Ag expression as measured by Western blot. The functional effects of T-Ag suppression were seen in cell culture in experiments that showed that the clonogenicity of cells measured in colony formation assays was also reduced. Thus our CRISPR/Cas9 system is able to target the T-Ag gene of JCV whether it was integrated into the cellular genome (HJC-2, BSB8) or whether it remains in a free episomal state entering the cell by transient transfection or by infecting viral genome (SVG-A). When we looked for InDel mutations in cellular genes with the highest homology to the three JCV target sequences using the SURVEYOR assay, none were found, indicating the specificity of targeting in this system.

As far as we are aware, this is the first report of CRISPR/Cas9 targeting JCV. The approach has been used for some other DNA viruses and retroviruses. The high risk human papillomaviruses HPV16 and HPV18 cause cervical carcinoma and other tumors resulting from the integration of HPV into the genome and expression of two HPV oncoproteins E6 and E7, which inactivate p53 and pRb respectively [39]. Kennedy et al [21] introduced Cas9 and E6- or E7-specific gRNAs into HeLa cells, a cervical carcinoma cell line which contains integrated HPV18 and reported inactivating mutations in E6 or E7 resulting in induction of p53 or pRb, resulting in cell cycle arrest and cell death. Similar results were obtained with the SiHa cervical carcinoma cell line, which contains integrated HPV16 indicating that the CRISPR/Cas9 approach is a potential effective therapy for HPV-induced tumors [21]. Similar results have been reported by other groups [40, 41].

A CRISPR/Cas9 approach has also been used against hepatitis B virus (HBV) [22]. HBV is a hepadnavirus with a small, circular DNA genome, which causes acute and chronic infections of the liver that can result in liver failure, cirrhosis, and hepatocellular carcinoma [42]. Chronic hepatitis B is associated with the persistence of closed circular episomal HBV DNA, which represents a barrier to viral eradication. Lin et al [22] identified active gRNAs that can act with Cas9 to reduce HBV in cell cultures. Introduction of these into mice using an adeno-associated viral vector also showed that they could reduce intrahepatic HBV load in vivo in a mouse HBV mouse model. The utilization of CRISPR/Cas9 against HBV has also been reported by Seeger and Sohn [43], Kennedy et al [44] and Zhen et al [45]. Epstein-Barr virus (EBV), a human DNA virus of the herpesvirus family that causes Burkitt’s lymphoma or nasopharyngeal carcinoma [46], has also been investigated by a CRISPR/Cas9 approach [47,48]. Cells from a Burkitt’s lymphoma with latent EBV infection showed a marked reduction in proliferation and decline in viral load after treatment with a CRISPR/Cas9 vector targeted to the viral genome [47]. Yuen et al [48] reported CRISPR/Cas9-mediated editing of EBV in human cells in several human epithelial cell lines latently infected with EBV including nasopharyngeal carcinoma. Another class of virus that is amenable to CRISPR/Cas9 manipulation is the retroviruses since they have a DNA intermediate in their life cycle. We have used CRISPR/Cas9 to target the HIV-1 LTR and to inactivate viral gene expression and replication in latently infected cells in culture [23] and similar approaches have been reported by other groups [49–51]. Permanent integration of HIV-1 into the host genome creates a reservoir of virus that is unreachable by current antiviral therapies and CRISPR/Cas9 was used to target the HIV-1 proviral genome. Specific targets were identified that completely excised integrated provirus. Moreover, the presence of HIV-1-specific gRNAs and Cas9 in cells prevents HIV-1 infection, i.e., CRISPR/Cas9 has both therapeutic and prophylactic potential [23]. Thus, CRISPR/Cas9 has tremendous potential against diseases caused by DNA viruses and retroviruses, particularly AIDS and certain cancers. Of note, about one-fifth of human cancers are thought to be caused by viruses and of the seven known human oncogenic viruses, six are DNA viruses or retroviruses [52].

In the case of JCV, we have demonstrated that the CRISPR/Cas9 system can be used to eliminate virus. This approach has the potential for both the removal of actively replicating virus in PML patients and the removal of asymptomatic persistent virus in individuals without PML but thought to be at risk for developing PML. Further development of this methodology for the clinic will require the use of up-to-date sophisticated approaches to gene delivery such as self-inactivating lentiviral vectors [53,54]. Also promising are adenoviral vectors [55] and adeno-associated virus vectors [56], which have the advantage that they do not integrate into the host DNA. Since PML is localized to the brain, intraparenchymal delivery to the brain may be applied.
